# Civilian Military Security Coordinators Coping with Frequent Traumatic Events: Spirituality, Community Resilience, and Emotional Distress

**DOI:** 10.3390/ijerph19148826

**Published:** 2022-07-20

**Authors:** Michael Weinberg, Adi Kimchy Elimellech

**Affiliations:** 1 School of Social Work, Faculty of Welfare and Health Sciences, University of Haifa, Haifa 3498838, Israel; bswadi85@gmail.com; 2 Sha’ar Menashe Mental Health Center, Haifa 3785000, Israel

**Keywords:** PTSD, stress, spirituality, community resilience, civilian security coordinators

## Abstract

Civilian military security coordinators are a unique kind of first responders. They live in communities that are close to the border and are responsible for the security of their community in routine and emergency situations until the arrival of the army or the police. Their role puts them at an elevated risk of experiencing emotional distress and developing PTSD. The present study, which was conducted in Israel following terror incidents over the year 2018, aimed to examine the relationships between spirituality and perceived community resilience, on the one hand, and PTSD symptoms and stress, on the other, among civilian military security coordinators. One hundred and thirteen (*n* = 113) civilian military security coordinators living up to 12.4 miles from the border who are routinely exposed to terror and other traumatic events completed demographic, spirituality, community resilience, PTSD, and stress questionnaires. Structural-equation-model analyses showed that spirituality was negatively associated with PTSD symptoms and stress. However, perceived community resilience was not associated with PTSD symptoms or stress. In addition, age was negatively associated with PTSD symptoms and stress. Financial situation was also negatively associated with PTSD symptoms and stress and incidence of exposure to terror and security threats was associated only with PTSD symptoms. Theoretical and practical implications are discussed.

## 1. Introduction

Exposure to traumatic events can cause a wide range of emotional distress [1] with post-traumatic stress disorder (PTSD) being the most researched consequence [2]. A unique group of people who are at a considerably high risk for trauma exposure are first responders, who are often exposed to war, terror, natural disasters, car accidents, death, injury, sexual violence, and other threats to health and security [3,4,5]. First responders are a diverse group of professionals, including firefighters, police, and paramedics, who are exposed to risk of death or severe injury. That stressful exposure distinguishes these jobs from other occupations [3,4,6]. Accordingly, first responders are also at greater risk of experiencing emotional distress and developing PTSD [3,4,7].

In Israel, over the past few years, an additional unique first-responder role has developed: the civilian military security coordinator. Communities near the border are often exposed to terrorist incidents. The role of civilian military security coordinator was established with the aim of providing an immediate initial response to protect citizens. The civilian military security coordinators are responsible for the security of their community in routine and emergency situations until the arrival of the army or police forces. As such, they fill military roles as a first responder to terror and other traumatic events, leading and commanding the community’s terror response force and providing medical assistance for those injured. These individuals also respond to car accidents, crime scenes, and incidents of domestic violence. Such routine confrontation with and exposure to trauma could lead to great risk and emotional distress.

However, to date, no studies have been conducted among civilian military security coordinators, to examine their emotional state and variables that could be related to their coping process. To that end, we examined the relationship between PTSD symptoms and stress and spiritualty, which reflects a universal dimension of human experience [8], and civilian military security coordinators’ perceptions of their community’s resilience, which reflect a more specific aspect of their daily work experience.

There is no single, well-defined, widely accepted definition of spirituality [9]. However, it is commonly accepted that spirituality is concerned with an individual’s inner life and is linked to a variety of human traits that aid in mental-health issues and well-being [10]. Spirituality represents an inner sense of wholeness and harmony with the environment and the community in which a person lives [11]. It captures the inherent motivation to search for meaning and significance in life [12]. Spirituality involves faith or acceptance of a belief system, as well as a personal search for meaning and purpose, encompasses an awareness of connection or relatedness to others, and/or is self-transcendent [13]. Spirituality has been found to be helpful for dealing with stressful situations [14,15]. In the context of trauma, numerous studies have found that spirituality is a significant resource for coping with emotional distress and PTSD symptoms [16,17,18,19].

Community resilience is an additional resource for coping with traumatic events that has attracted attention in recent years [20]. Community resilience reflects the community’s ability to plan for, recover from, and adapt to stressors [21,22]. Although there is no consensus as to what community resilience is, how it should be defined, or what its core characteristics are [23], community resilience is generally perceived as a multidimensional construct encompassing basic physical needs and providing protection. It includes the amount of stress that a system can absorb, the degree to which the system is capable of self-organization, and the degree to which can build and increase capacity for learning and adaptation [24,25]. Community resilience reflects several different factors, including social resources, social relationships, leadership, community history, and community culture [26]. In general, it includes the way that people relate to each other, as well as interpersonal and collective resources and capacities [27,28]. Various studies have examined the importance of community resilience for coping with trauma and stress, demonstrating its important role in this area. For example, Kimhi [28] reviewed the associations among individual, community, and national resilience and noted the positive correlations between them. In that work, demographic variables such as older age and higher levels of religiosity positively predicted resilience and all three levels of resilience predicted individual well-being and successful coping with potential traumatic events. In a study conducted among 294 participants who had suffered from Hurricane Katrina and the Deepwater Horizon oil spill, community resilience was found to be positively related to psychological resilience. In addition, community resilience was also related to lower levels of depressive symptoms through the mediating variable of psychological resilience. This finding demonstrates the importance of community resilience for improved mental-health outcomes [29]. A recent study [30] conducted among 2246 individuals who had suffered from disasters such as earthquakes, typhoons, flooding, and fires found that community resilience can serve as a community-level coping resource in times of collective crisis, as well as a protective factor that facilitates recovery among disaster victims. These findings highlight the importance of psychological resilience in long-term disaster recovery and imply that long-term recovery efforts should address factors associated with both psychological and community resilience to improve mental health outcomes. As civilian military security coordinators are responsible for the safety of their communities and considering that personal resources are associated with PTSD symptoms and stress, the present study strived to examine the relationship between civilian military security coordinators’ perceptions of their community’s resilience, their personal spirituality, and their emotional distress. Accordingly, two hypotheses were postulated:

Spirituality will be negatively associated with civilian military security coordinators’ PTSD symptoms and stress.

Civilian military security coordinators’ perceptions of the resilience of their community will be negatively associated with their levels of PTSD symptoms and stress.

## 2. Materials and Methods

**Figure 1:**
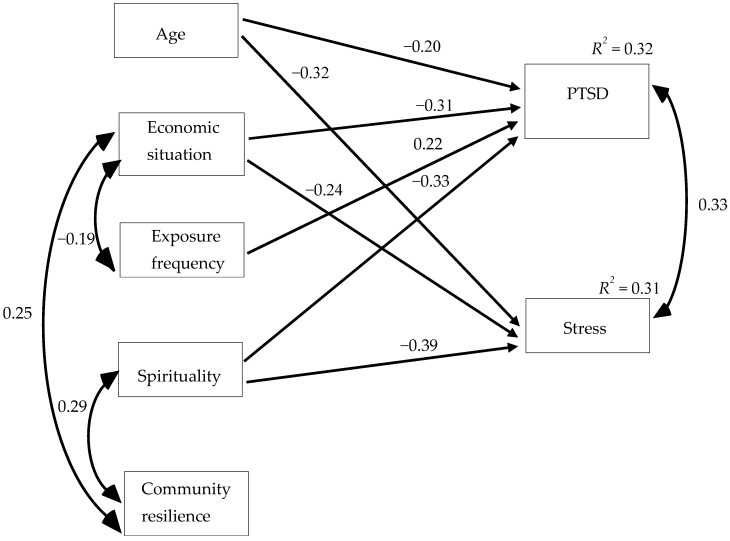
The associations between age, economic situation, frequency of exposure, spirituality, perceived community resilience, PTSD, and stress.

The data were collected using published survey scales and was examined in three stages. In the first stage, we used IBM SPSS Statistics 27 to examine demographic and general data regarding the participants (Table 1). In the second stage, we used that same program to examine the means of the research variables and their standard deviations, as well as correlations between those variables and performed independent-sample t-tests (Table 2 and Table 3). Finally, in the third stage, we used AMOS (version 25, IBM: Armonk, NY, USA) to conduct structural-equation modeling, to examine the study model and hypotheses (Figure 1).

### 2.1. Measures

**Demographic questionnaire.** This questionnaire covered variables such as age, education, financial situation, work experience, and exposure to trauma.

**Spirituality.** Spirituality was assessed using the Spirituality Assessment Scale (SAS; [31]). The SAS includes 28 items that are each rated on a 6-point scale ranging from 1 (*strongly disagree*) to 6 (*strongly agree*). Higher scores reflect higher levels of spirituality. In total the score range for the scale was 28–168. The present study used a translated version of the SAS that has been used in previous studies, in which it demonstrated high internal-consistency [19]. The Cronbach’s alpha internal-consistency coefficient for the SAS scale was 0.92.

**Community resilience.** Perceived community resilience was examined using the community-resilience scale developed by Kimhi and Shamai [26]. The scale was originally developed in the local language thus the present study used the original scale. The scale includes 10 items examining social resources, social relationships, leadership, community history, and community culture. The items are each rated on a 5-point scale ranging from 1 (*strongly disagree*) to 5 (*strongly agree*). Higher scores reflect higher levels of community resilience. The score range for the scale was 10–50. The Cronbach’s alpha internal-consistency coefficient was 0.88.

**PTSD symptoms.** PTSD symptoms were evaluated using the PTSD Symptom Levels Scale (PSL) questionnaire compiled by Gil and colleagues [32]. The PSL was originally developed in the local language. As such the present study used the original scale. The PSL is a 20-item, self-report questionnaire that aims to assess the level of PTSD symptoms complying with the criteria for PTSD diagnosis as presented in the *DSM-5*. The severity of each item is rated on a 4-point Likert scale ranging from 1 (*not at all* or *once a week*) to 4 (*five times a week or more*). Higher scores reflect higher levels of PTSD symptoms. Accordingly, the score range for the scale was 20–80. The Cronbach’s alpha internal-consistency coefficient was 0.94.

**Stress.** Stress was assessed using the Perceived Stress Scale (PSS); Cohen and colleagues [33]. The PSS scale is a 10-item, self-report questionnaire aimed at assessing levels of perceived stress over the past month. Each item relates to situations in one’s life that are appraised as stressful. The severity of each item is rated on a 5-point Likert scale ranging from 1 (*never*) to 5 (*very often*). Higher scores represent higher levels of stress. The score range for the scale was 10–50. The present study used a translated version of the PSS scale that has been used in previous studies, in which it demonstrated high internal-consistency [19]. The Cronbach’s alpha internal-consistency coefficient was 0.83.

### 2.2. Procedure

Following the approval of the University ethics committee, all 229 civilian military security coordinators who are members of the national association of the Israeli civilian military security coordinators, were approached and asked to take part in the study. At that time, they were also presented with explanations of the importance and goal of the study. Those who agreed to participate in the study signed the informed consent form and were then given the study questionnaire. Initially, 169 coordinators agreed to take part in the study and 113 fully completed the study questionnaire. All participants voluntarily completed the questionnaires. Assistance was limited to clarifying instructions and making sure that all items were completed. No compensation was offered for participation in the study.

## 3. Results

The sample consisted of 113 participants (*n* = 113) living up to 12.4 miles from the border. All participants were male. As shown in Table 1 regarding the sociological characteristics of the participants the average number of years of education among the participants was 13.93 years (*SD* = 2.26). Twenty of the participants (17.7%) reported perceiving their financial status as high, 84 (74.3%) reported an average financial status, and nine (8.0%) reported a low financial status. The average age was 43.68 (*SD* = 9.90). In terms of exposure to traumatic events aside from their work as civilian military security coordinators, 26 (23%) reported that they had not been exposed to any other traumatic events and 87 (77%) reported that they had been exposed to such events, including physical assault (8%), combat (26.5%), terror attacks (24.8%), serious sickness or disease (1.8%), and other unspecified trauma (15.9%). Twenty-four participants (21.2%) had up to 2 years of experience as civilian military security coordinators, 51 (45.1%) had up to 10 years of experience, and 38 (33.6%) had over 10 years of experience. The average number of incidents of exposure to terror/security threats over the previous 3 months was 7.41 (*SD* = 11.92).

As shown in Table 2, negative correlations were found between PTSD and spirituality (*r* = −0.35, *p* < 0.01) and between PTSD and perceived community resilience (*r* = −0.21, *p* < 0.05). Negative relationships were also found between stress and spirituality (*r* = −0.39, *p* < 0.01) and between stress and perceived community resilience (*r* = −0.21, *p* < 0.05). A positive correlation was found between PTSD symptoms and stress (*r* = 0.55, *p* < 0.001). In addition, we also examined the relationships between demographic and general variables, on the one hand, and PTSD symptoms and stress, on the other.

As shown in Table 3 regarding the relationship between demographic variables and PTSD and stress, age was found to be negatively correlated with both PTSD symptoms and stress (*r* = −0.30, *p* < 0.01; *r* = −0.35, *p* < 0.01, respectively). Negative correlations were also found between economic situation and PTSD symptoms and stress (*r* = −0.37, *p* < 0.01; *r* = −0.26, *p* < 0.01, respectively). Positive correlations were found between exposure to terror and security threats over the past 3 months and PTSD symptoms and stress (*r* = 0.39, *p* < 0.01; *r* = 0.26, *p* < 0.01, respectively). The number of years of experience as a civilian military security coordinator was negatively correlated with stress, but not with PTSD symptoms (*r* = −0.22, *p* < 0.05; *r* = −0.12, *p* = *ns*, respectively) and no significant correlations were found between level of education and PTSD symptoms and stress. In addition, we also examined if there were differences in levels of PTSD symptoms and stress between participants who reported exposure to traumatic events aside from their professional role and those who reported not having experienced such exposure. Independent sample *t*-tests demonstrated that there were no significant differences in levels of PTSD symptoms (M = 28.39, SD = 9.45; M = 25.64, SD = 7.87; t(95) = −1.24, *p* = *ns*) or levels of stress ((M = 24.93, SD = 5.85; M = 25.42, SD = 7.01; t(105) = 0.344, *p* = *ns*) between those two groups of participants.

Finally, structural-equation modeling was conducted with AMOS (version 21, IBM: Armonk, NY, USA) to examine how PTSD symptoms and stress were associated with the study variables (Figure 1). At first, the research model included all possible paths between the study variables. However, this type of model does not allow for degrees of freedom, and therefore the fit of the model to the empirical data could not be estimated. Therefore, to obtain the most parsimonious model and estimate how well that model fit the empirical data, nonsignificant paths were removed. A model in which χ^2^/df is ≤2, CFI and NNFI are greater than 0.90, and RMSEA is between 0.00 and 0.08 is deemed acceptable [34]. The model demonstrated good fit: χ^2^(10) = 11.799, *p* = 0.299, χ^2^/df = 1.17, NNFI = 0.90, CFI = 0.98, TLI = 0.95, RMSEA = 0.04.

As shown in Figure 1, spirituality was negatively associated with both PTSD symptoms (β = −0.33, *p* < 0.01) and stress (β = −0.39, *p* < 0.001). However, perceived community resilience was not associated with PTSD symptoms or stress. In addition, age was negatively associated with PTSD symptoms (β = −0.20, *p* < 0.01) and stress (β = −0.32, *p* < 0.001), financial situation was negatively associated with PTSD symptoms (β = −0.31, *p* < 0.001) and stress (β = −0.24, *p* < 0.01), and incidence of exposure to terror and security threats was associated only with PTSD symptoms (β = 0.22, *p* < 0.01). The study model significantly explained 32% of the variance of PTSD symptoms and 31% of the variance in stress.

## 4. Discussion

The main goal of this study was to examine the relationships between spirituality and perceived community resilience and emotional distress among civilian military security coordinators, as reflected in their PTSD symptoms and stress levels. In accordance with our research hypothesis, higher levels of spirituality were associated with lower levels of PTSD symptoms and stress. This finding further demonstrates the importance of spirituality as a key resource for dealing with trauma [16,17,18,19]. Furthermore, since spirituality has rarely been examined in the context of first responders, this finding adds an additional perspective regarding the potential impact of spirituality as an internal resource for civilian military security coordinators and other first responders faced with a wide range of types of emotional distress, including PTSD symptoms and general stress. In addition, spirituality reflects a universal dimension of human experience, search for meaning, meaning in life and wholeness, harmony with the community, and connection or relatedness to others [8,11,12,13]. It is possible that civilian military security coordinators perceive their occupation as meaningful, significant, and relevant to different aspects of the well-being and safety of their community. For that reason, among this population, higher levels of spirituality might be associated with lower levels of emotional distress. Surprisingly and contrary to our second hypothesis, the study model revealed no significant relationships between community resilience as perceived by the civilian military security coordinators and their own levels of PTSD symptoms and stress. Notably, this study is the first to examine civilian military security coordinators. Civilian military coordinators include and reflect components of several roles related to the security and safety of civilians, including policing, community policing, and military roles. In this context, it has been suggested that police forces should seek direction from their customers: the community [35]. Taking into account that the coordinators are civilians who are part of the community they serve, it is possible that they perceive that their customers (i.e., the members of their community) expect them to, first and foremost, protect their physical safety, as opposed to prioritizing on other issues. Therefore, the coordinators might be less sensitive to community resilience. Following this notion, it has been argued that community resilience is an amorphous concept [23] and, in the context of communities facing chronic problems, resilience might be a vague and tangential notion [36]. Thus, it is possible that the circumstances of living in close proximity to the border and suffering from continuous traumatic events are experienced as chronic problems by the civilian military coordinators, which might increase vagueness in the context of community resilience. However, this assumption should be made with caution, as community resilience has been measured in the past among communities that were suffering from intense rocket-fire [26].

On the other hand, it is also possible that first responders may often develop strategies to detach themselves from emotional states, in order to cope with challenging tasks [37,38]. Accordingly, it could be that as civilian military coordinators focus on their job, as part of their coping process, they are less aware of the community resilience and focus more on their daily conduct. It should be noted that the levels of PTSD among the coordinators were not particularly high. These findings could possibly be explained in line with Solomon and colleagues’ study [39], which was conducted among rescue workers whose work involved body handling. That work found relatively low levels of psychiatric symptomatology among those rescue workers. They suggested that motivation and self-selection factors might have contributed to these low levels. Accordingly, it is possible that coordinators’ unique position in their community, their motivation, self-selection, and personal traits such as spiritualty may be associated with their coping processes and emotional state.

This finding might suggest that there is a difference between community resilience as perceived by community members, which has been shown to be an important resource for coping with trauma [28,29,30], and community resilience as perceived by civilian military security coordinators. Nevertheless, as community resilience can play an important role in coping with trauma, it would be worthwhile to further examine additional ways in which community resilience could be associated with coping with emotional distress among civilian military security coordinators.

As the study model demonstrates, when referring to civilian military security coordinators, personal characteristics should also be taken into account. As can be expected, exposure to more traumatic events was related to higher levels of PTSD symptoms. Interestingly, this relationship was found only for PTSD and not for general stress. This finding could further emphasize the uniqueness of PTSD as related to trauma encounters and different from general stress [19,40]. Although age is often considered to be a risk factor for PTSD symptoms and, among first responders, age has also been shown to be related to higher levels of emotional distress [41,42], being older could also be related to lower levels of emotional distress among first responders [43]. The study model used in this work demonstrates that age is associated with lower levels of PTSD symptoms and stress. It is possible that within the context of being a first responder, such as a civilian military security coordinator, age can have an advantage, in terms of how the coordinators are viewed by their communities. Age could be related to more years of experience, greater prestige, and more personal professional confidence, all leading to better coping.

## 5. Conclusions

The findings of the study have important implications. When helping civilian military security coordinators to cope with PTSD and stress, we suggest that special attention and programs address spirituality as it is associated with lower levels of both PTSD symptoms and stress. We also recommend that attention be paid to the number of traumatic events to which the coordinators are exposed, since greater exposure is associated with higher levels of emotional distress. Although the study model did not demonstrate a relationship between community resilience and emotional distress, it is worthwhile to encourage civilian military security coordinators to be aware of their community’s resilience and to further re-examine possible relationships between community resilience and emotional distress.

Several limitations of this work should be noted. The cross-sectional nature of this study precludes the identification of any causal relationships. In addition, although this is the first study to be conducted among civilian military security coordinators, the relatively small sample requires that we use caution when generalizing these findings. Nevertheless, despite these limitations, this study makes an important theoretical and practical contribution to our understanding of the relationships between spirituality and community resilience and PTSD symptoms and stress.

## Figures and Tables

**Table 1:** Sociological characteristics of the population.

		Frequency	Percentage
Financial status	Low	9	8.0
	Average		74.3
	High	20	17.7
Exposure to additional traumatic events	no exposure	26	23
	physical assault	9	8
	Combat	30	26.5
	terror attacks	28	24.8
	serious sickness/disease	2	1.8
	unspecified trauma	18	15.9
Years of experience as coordinators	up to two years	24	21.2
	up to ten years	51	45.1
	over ten years	38	33.6
		**Mean**	**SD**
Age		43.68	9.90
Years of education		13.93	2.26
exposure to terror/security events over the previous 3 months		7.41	11.92

**Table 2:** Means, standard deviations, and correlations between PTSD, stress, community resilience, and spirituality.

		Mean (SD)	PTSD	Stress	Community Resilience	Spirituality
1.	PTSD	27.76 (9.14)		0.55 **	−0.21 *	−0.35 **
2.	Stress	25.04 (6.11)			−0.21 *	−0.39 **
3.	Community resilience	36.45 (7.21)				0.26 *
4.	Spirituality	115.45 (21.02)				

* p < 0.05. ** p < 0.01.

**Table 3:** Correlations and differences between demographic variables and PTSD and stress.

		PTSD		Stress	
1.	Age	−0.30 **		−0.35 **	
2.	Economic situation	−0.37 **		−0.26 **	
3.	Exposure to terror and security threats over the past 3 months	0.39 **		0.26 **	
4.	Years of experience as a civilian military security coordinator	−0.12		−0.22 *	
5.	Years of education	−0.12		−0.03	
		*Mean*	*SD*	*Mean*	*SD*
6.	Exposure to traumatic events aside from civilian military security coordinator exposure	28.39	9.45	24.93	5.85
7.	No exposure to traumatic events aside from civilian military security coordinator exposure	25.64	7.87	25.42	7.01

* *p* < 0.05. ** *p* < 0.01.

## Data Availability

The data presented in this study are available on request from the corresponding author.
